# Mapping patterns of complementary and alternative medicine use in cancer: An explorative cross-sectional study of individuals with reported positive "exceptional" experiences

**DOI:** 10.1186/1472-6882-8-48

**Published:** 2008-08-08

**Authors:** Johanna Hök, Carol Tishelman, Alexander Ploner, Anette Forss, Torkel Falkenberg

**Affiliations:** 1 Karolinska Institutet, Dept of Neurobiology, Care Sciences and Society; Division of Nursing, Unit for Studies of Integrative Care, 23 300, SE-141 83 Huddinge, Sweden; 2 Karolinska Institutet, Dept of Learning, Informatics, Management and Ethics, Medical Management Center, SE-171 77 Stockholm, Sweden; 3 Karolinska Institutet, Dept of Medical Epidemiology and Biostatistics, PO Box 281, SE-171 77 Stockholm, Sweden; 4 Research and Development Unit, Stockholms Sjukhem Foundation, SE-112 35 Stockholm, Sweden; 5 University of Manchester School of Nursing, Midwifery and Social Work, University Place, Oxford Road, Manchester M13 9PL, UK; 6 State University of New York, Dept of Philosophy, Technoscience Research Group, Stony Brook, USA

## Abstract

**Background:**

While the use of complementary and alternative medicine (CAM) among cancer patients is common and widespread, levels of commitment to CAM vary. "Committed" CAM use is important to investigate, as it may be associated with elevated risks and benefits, and may affect use of biomedically-oriented health care (BHC). Multiple methodological approaches were used to explore and map patterns of CAM use among individuals postulated to be committed users, voluntarily reporting exceptional experiences associated with CAM use after cancer diagnosis.

**Method:**

The verbatim transcripts of thirty-eight unstructured interviews were analyzed in two steps. First, manifest content analysis was used to elucidate and map participants' use of CAM, based on the National Center for Complementary Medicine (NCCAM)'s classification system. Second, patterns of CAM use were explored statistically using principal component analysis.

**Findings:**

The 38 participants reported using a total of 274 specific CAM (median = 4) consisting of 148 different therapeutic modalities. Most reported therapies could be categorized using the NCCAM taxonomy (n = 224). However, a significant number of CAM therapies were not consistent with this categorization (n = 50); consequently, we introduced two additional categories: *Spiritual/health literature *and *Treatment centers*. The two factors explaining the largest proportion of variation in CAM usage patterns were a) number of CAM modalities used and b) a category preference for *Energy therapies *over the categories *Alternative Medical Systems *and *Treatment centers *or vice versa.

**Discussion:**

We found considerable heterogeneity in patterns of CAM use. By analyzing users' own descriptions of CAM in relation to the most commonly used predefined professional taxonomy, this study highlights discrepancies between user and professional conceptualizations of CAM not previously addressed. Beyond variations in users' reports of CAM, our findings indicate some patterns in CAM usage related to number of therapies used and preference for different CAM categories.

## Background

Use of complementary and alternative medicine (CAM) among people with cancer has been found to be common and widespread with estimates ranging between 7–64% [1]. Apart from probable differences in CAM utilization in different settings and countries, this variation is partially related to differences in the instrumentalization of CAM definitions [1]. The wide spectrum of therapies often considered as within the CAM domain is indicated for example in the broad definition by the Cochrane Collaboration, which includes "all such practices and ideas self-defined by their users as preventing or treating illness or promoting health or well-being" if they are not part of the "politically dominant health system of a particular society or culture" at the time [2]. The growing number of CAM modalities subjected to efficacy studies is yet another indication of the breadth of this area. Studies are also called for to investigate potential risks in CAM use, including side-effects and interactions between CAM preparations and BHC treatments.

Considering the frequency of CAM use and the wide spectrum of therapies included, it is not surprising that studies suggest that there may be important differences among CAM users, e.g. with regard to types of CAM used and how therapies are combined [3]. A number of CAM taxonomies have been proposed to distinguish CAM use by type of therapy. Tataryn [4] suggests categorization according to basic assumptions of health and disease underlying each therapy. Jones [5] on the other hand, argues that it is more clinically relevant to categorize CAM according to primary mode of therapeutic action. In line with Jones' suggestion, the influential N.I.H. National Center of Complementary and Alternative Medicine (NCCAM) in the U.S. [6], describes CAM in five categories: *Alternative Medical Systems*; *Mind and Body Interventions*; *Biologically Based Therapies*; *Manipulative and Body Based Therapies; *and *Energy Therapies*, which is further distinguished into the subcategories *Biofield therapies *and *Bioelectromagnetic-based therapies*. Many CAM utilization studies have used this framework when assessing CAM use.

Several studies have found that cancer patients utilize therapies from all NCCAM categories, often using multiple CAM therapies during their disease trajectory [1,7,8]. In their study of women with breast cancer, Balneaves et al [9] further distinguished "committed" CAM use, which they defined as use of a large number of CAM therapies, coupled with extensive time, energy and financial resources spent on this use. With consideration given the dedicated use and the large number of CAM therapies involved, committed CAM use may be particularly relevant to further investigate since it may be associated with elevated risks [10] and unclear benefits. The possibility for interactions makes it important to investigate not only the number of therapies used, but also relationships among them.

The data presented here provides a complement to existing literature by using detailed unstructured narratives from CAM users as a basis for exploring CAM use. Multiple methodological approaches were used to map and explore patterns of CAM use, including use of therapies across different categories, in a self-selected group hypothesized to represent one type of committed use. This material derives from the Swedish portion of a Nordic collaboration, investigating exceptional experiences around CAM and cancer [11], with other reports delving further into the experience of the participants [12].

## Methods

The data presented here are drawn from a larger project exploring different stakeholder perspectives of exceptional CAM experiences, inspired by a similar program by National Cancer Institute (N.C.I.), N.I.H., U.S. [13]. We used an inductive approach to allow insight into individuals' perspectives of exceptional experiences in connection to CAM use, with a critical incident design to locate "extreme" and "extraordinary" accounts [14].

### Data collection

After approval by the Karolinska Institutet research ethics review board, mass media was used to invite reports about CAM use in connection to experiences perceived as exceptional, in the sense of unexpected or unusual improvement or deterioration of the health of people with cancer. No further predefinition was provided for what was considered exceptional or for what was considered a CAM therapy, in order to explore stakeholders' own conceptualizations.

Thirty-eight people with cancer were interviewed between April 2004 and November 2005. Twenty-eight participants contacted the researchers actively themselves, while the remaining 10 cases were first reported by a CAM provider after receiving patient consent. We conducted open interviews that generally lasted one to three hours, to encourage participants' accounts of their CAM use and illness experiences. Thirty-six participants consented to the interview being audio-recorded and later transcribed verbatim. Detailed interview notes were taken after consent from two participants who were uncomfortable with audio-recording. All study participants were given fictitious names.

### Descriptive Analysis

Following principles of manifest content analysis [15,16], all CAM mentioned by the participants in interview transcripts, e-mails and letters were identified and coded using the qualitative data analysis program NVivo [17]. All therapies defined by participants as utilized as complements or alternatives to BHC treatments in connection to their cancer were first sorted into one of the five NCCAM categories. Fifty accounts did not fit into any of the existing NCCAM categories, despite participants' narrative reports of their therapeutic nature. After further analysis of these accounts, two additional categories were formulated, *Spiritual/health literature *and *Treatment centers combining CAM and BHC *(referred to also as *Treatment centers*). This resulted in seven categories of CAM therapies. A CAM educator external to this project later confirmed categorization. Inter-rater reliability was high, with only two inconsistencies in categorization among the 148 different therapies.

Demographic and disease characteristics were extracted from the interview data and are presented by minimum (min) and maximum (max) values, with inter-quartile ranges (IQR) shown.

### Explorative statistical analysis

Principal component analysis was used to explore correlations between usage of CAM categories. New variables (principal components, PCs), were introduced to reduce the dimensionality of the usage pattern while retaining as much as possible of the variation in the original data. PCs are computed as weighted sums of the original variables, where the weights of the original variables are referred to as loadings. By definition, the first PC expresses the greatest amount of variation in the data, the second PC the next largest amount, and so on.

By applying the weights of the original variables, i.e. the loadings, to the values observed for each participant, we also computed the scores along each PC for these participants [18]. Calculation of loadings was based on correlations between therapy counts in the seven CAM categories. Using correlations here corresponds to a standardization of the observed therapy counts to the mean of zero and standard deviation one across all participants. In this manner, all categories have the same weight in calculating the loadings. The use of correlations avoids biasing the PC analysis towards categories comprising more therapy modalities at the expense of categories with less reported modalities [18].

Bootstrap confidence intervals were computed for the loadings of the original variables to allow estimation of standard errors and confidence intervals without strong parametric assumptions [19]. The bootstrapped confidence intervals served as guidelines for the selection of the number of PCs to be retained, and for the interpretation of the loadings. No formal inference to a larger underlying population is intended in this exploration.

Two types of usage patterns indicated by the first two PCs were analyzed. One such usage pattern was calculated based on the magnitude and sign of the contribution of each CAM category to the PCs. A second pattern was calculated based on each user's score on the retained PCs. These calculations resulted in individual usage patterns, displayed in a plot to identify groups of participants with similar usage profiles. To aid the visual impression, an explorative k-means clustering analysis of the scores was performed. The number of clusters was chosen to maximize a measure of average separation between members of different clusters (silhouette width as described in [20]).

## Results

### Demographic and disease characteristics of participants

Tables 1 and 2 present sample characteristics as reported by the study participants, with resulting gaps in information on occupational status and level of education. At the time of interview, the study sample ranged in age between 36 and 85 years (median = 55, IQR = 48–63 years) and was composed primarily of women, with half the participants living with a partner. The dominance of women is reflected in the diagnostic pattern of the sample, with 24 of the 38 participants reporting primary breast or gynecologic tumors. Fifteen participants reported having metastasized cancer. The time between first cancer diagnosis and time of interview ranged from one to 32 years (median = 5 years, IQR = 1–13 years).

**Table 1:** Participant characteristics.

Characteristics	Frequency (n = 38)
Age	Median 55 years (min = 36, max = 85)

≤ 40 years	3
41–50 years	9
51–60 years	13
61–70 years	6
>70 years	5
Age unknown	2
Sex	
Female	31
Male	7
Marital status	
Married or common-law	19
Divorced/Separated/Widowed/Single	15
Unknown	4
Occupational Status	
Working full-time	7
Working part-time	2
On sick-leave	7
Pension	9
Unknown	13
Education	
College education	20
Elementary school + High School	4
Unknown	14

**Table 2:** Reported disease characteristics.

Reported disease characteristics	Frequency (n = 38)
Breast	17
Gynecological (women)	7
Stomach, Colon and Rectum	4
Lymphatic leukemia	2
Lung	2
Prostate	2
Other sites	4
Metastasized disease	15
Median time since 1^st ^cancer diagnosis (years)	5 years

### CAM use in relation to BHC treatment and disease characteristics

Seventeen participants reported completing BHC treatments according to medical recommendation, six participants did not discuss this issue, and 15 of the 38 participants said they had not completed recommended BHC treatments. In 14 accounts this decision was framed as a choice either against or without BHC medical advice, and in one case as a decision made in conjunction with BHC advice. Participants who reported completing BHC treatment used a median of three different CAM therapies (min = 1, max = 20, Q_1 _= 2, Q_3 _= 7) from a median of two different CAM categories (min = 1, max = 7, Q_1 _= 2, Q_3 _= 4.5), while participants who did not complete BHC treatments reported using a median of seven different CAM therapies (min = 1, max = 26, Q_1 _= 4, Q_3 _= 12) from a median of four CAM categories (min = 1, max = 7, Q_1 _= 3, Q_3 _= 5).

Fifteen participants described having metastasized cancer, while 23 reported local disease. Participants reporting metastasized cancer used a median of seven CAM therapies (min = 3, max = 26, Q_1 _= 3, Q_3 _= 12) from a median of five categories (min = 1, max = 6, Q_1 _= 3, Q_3 _= 6), whereas those who reported a local cancer used a median of three CAM therapies (min = 1, max = 22, Q_1 _= 2, Q_3 _= 7) from a median of two categories (min = 1, max = 7, Q_1 _= 2, Q_3 _= 4).

### Description of CAM reports

The 38 participants diagnosed with cancer described using a total of 274 CAM therapies consisting of 148 different therapeutic modalities (Table 3). Between one-26 different CAM therapies were reported as utilized by participants (median = 4, IQR = 1–8). CAM treatments across all categories were used throughout the cancer trajectory. Thirty-two participants reported being in contact with CAM providers, with a median of one CAM provider utilized per participant (min = 1, max = 7, Q_1 _= 1, Q_3 _= 2). The remaining six participants reported using only self-care CAM modalities.

**Table 3:** CAM described by participants and sorted into seven CAM categories.

**NCCAM CATEGORIES**	Total number of therapies reported	Number of individuals reporting therapies
ALTERNATIVE MEDICAL SYSTEMS antroposophic medicine (6), homeopathy (3), traditional Chinese medicine	10	10

MIND-BODY INTERVENTIONS painting (6), music, dance, sculpturing, counselling (6), support groups, mental practice (6), relaxation techniques (2), eurythmy (3), gestalt therapy, bonitology (2), kinesiology, prayer (3), meditation-various types (9), family constellations, visualization (3), rehabilitation program, rosen method body work	50	23

BIOLOGICALLY-BASED THERAPIES aloe vera (2), angelica, antioxidants (5), apis, ayurvedic preparations, birch ash (2), blutsaft, cayenne pepper, cetraria, chalk, charchole, chinese herbal medicine, cypress, coffee enema, dendrite cell treatment (2), ecomer, edta, enzymes (2), field horsetail, fish oil, garlic (2), geranium, ginger, ginseng, helixor (2), iceland lichen, inhalation mixture-chamomile, peppermint and lemon balm, iscador (14), juniperberry, kan yang, lactase enzyme, lavender, lemon concentrate (2) lemon grass, lemon balm, linseed bandage, lycine, magnesium (2), marjoram, micro-algae, mung bean sprouts (2), new castle virus (2), nouni (2), olibanum, ozone therapy (2) quercetin, pankreon, probion, proline, proteas, radish, raw food diet, rosemary, sage (2), sandal wood, saw palmetto, selen (2), shark liver oil, silica, silymarin, silver, sodium ascorbate, sodiumselen respond selen, sulfur, supergreens, THX, valerian root, vegan diet, vitamin A, vitamin b, vitamin C (3), vitamin D, vitamin e (5), walnut supplements, wheat grass juice, yarrow, zinc (2)	115	27

MANIPULATIVE AND BODY BASED THERAPIES acupuncture (3), chiropractic care, feldenkreis, fever baths, herbal baths, local and whole body hyperthermia (2), stretching, lymph massage (2), alternative surgical procedure, soft tissue massage	14	12

ENERGY THERAPIES Biofield therapies: healing (10), qi gong (4), tai chi (2), yoga (3), reflexology, color therapy, homeopathic remedies (gold, arsenic, barium-iodate, viscum/mesenchym comp, conium maculatorn) Bioelectromagnetic-based therapies: ECT-laser (3), frequency medicine (2), magnetic field therapy (3), plasma lamp therapy	35	21


**EMPIRICALLY DERIVED CATEGORIES**		

SPIRITUAL/HEALTH LITERATURE A Course in Miracles-author unspecified, Bays Brandon – The Journey (2), Chopra Deepak – Perfect Health, Ehdin, Sanna – The Self-Healing Human (4), Gawler Ian – You can conquer cancer, Hamer Gerhard – The New Medicine (3), Pollak Kay – Att välja glädje [only in Swedish], Hayes Louise – no specific book, Alexander Marcus – Kvantmänniskan [in Swedish], Preben Maria – no specific book, Moss Ralph-Cancer & CAM information, Shine Betty – Mind to Mind, Siegel Bernie – Love, Medicine and Miracles (6), Sai Baba – no specific book, Simonton, Carl – Getting Well Again, Stern Bengt – Feeling bad is a good start (3), Walsch Donald – Conversations with God	31	15

TREATMENT CENTERS Centro Antroposophico – Antroposophic center, Spain; Furusjön – Health retreat, Sweden (2); Humlegården – Alternative Clinic for cancer patients, Denmark (2); Lustgården – Rehabilitation unit for cancer patients, Sweden, Mösseberg-rehabilitation for cancer patients, Sweden (2), Vidarkliniken – Antroposophic hospital, Sweden (10), TCM hospital combining TCM and BHC, Germany	19	15

Number of participants reporting a certain CAM is indicated in parentheses (if more than one).

#### Categorization of therapies according to the NCCAM system

The category *Biologically-based therapies *was the most commonly described CAM category, reported by 27 of 38 participants. This category comprised 77 different therapies with Iscador^®^, an injectable extract of mistletoe, most common (n = 14). *Mind-body interventions *was the second most common CAM category reported by 23 participants, with meditation most frequently reported (n = 9). Painting therapy, mental training and counseling were each reported by several participants. Twenty-one participants reported using *Energy therapies*. The therapies within this category were sorted into the NCCAM's sub-categories; *Biofield therapies *including healing, qi gong and yoga and *Bioelectromagnetic-based therapie*s including magnetic field therapy and laser therapy. Healing, used by 10 participants, was the most common therapeutic modality in this category. The category *Alternative medical systems *was reported by 10 participants and consisted of Antroposophic medicine (n = 6), Homeopathy (n = 3) and Traditional Chinese Medicine (n = 1). The category *Manipulative and body-based therapies *was reported to be used by 12 individuals, with acupuncture, hyperthermia and lymph massage each used by two participants.

#### Empirically derived categories

The empirically-derived category *Spiritual/health literature *consisted predominantly of inspirational literature about CAM and cancer in a broad context, with the book "Love, Medicine and Miracles" by Bernie Siegel [21] referred to by the largest number of participants (n = 7). In total, 15 participants provided reports categorized under this heading. A second empirically-derived category consisted of seven different *Treatment centers combining CAM and BHC*, which could be placed along a continuum with varying levels of integration between different therapies from use of psychosocial interventions in a BHC setting, to integration of BHC and CAM treatment. Fifteen participants reported such use, most frequently referring to one antroposophic hospital (n = 10), where health care providers are licensed in both BHC and antroposophic medicine. Reports within this category focus on the environment of the centers, as well as encounters with staff and other patients, rather than on specific therapeutic modalities.

### Patterns of CAM use

We have thus described CAM use in seven categories. Most participants described using therapies from several categories, with a median of three CAM categories (min = 2, max = 7, Q_1 _= 2, Q_3 _= 5). The number of specific therapies reported for each category varied widely between CAM categories. The greatest variety of specific therapies used within the same category was found in regard to *Biologically-based therapies*.

**Figure 1:**
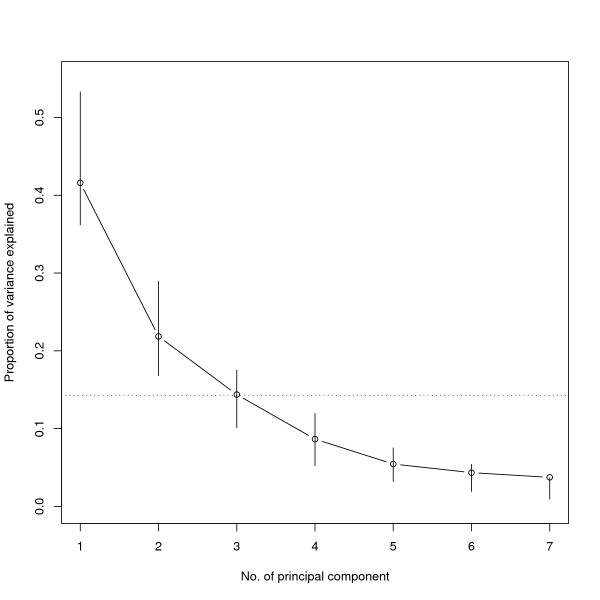
**Scree plot showing the proportion of variance explained by consecutive principal components (PCs)**. Bootstrapped 95% confidence intervals are shown as vertical lines. The dotted horizontal reference line indicates the proportion of variance explained by one of the underlying variables (i.e. category counts).

In the explorative PC analysis we found that PC1 to PC3 accounted for 42%, 22% and 14%, respectively, of the underlying data. We interpreted the curve of percentages as steep between PC1 and PC2, and as flattening out after PC2 (as shown in Figure 1). This motivated retaining PC1 and PC2 [18], which together explained over 63% of the variability of the scaled usage counts.

As shown in Table 4, all loadings for PC1 were positive. We interpret this PC as a weighted average of the number of treatments reported within each category. PC2 has both positive and negative loadings (Table 4), with the three categories with loadings significantly different from zero (based on 95% confidence intervals): *Alternative medical systems *and *Treatment centres *significantly negative and *Energy therapies *significantly positive. We interpret PC2 as indicative of a preference for CAM categories along a continuum, indicated by the sign (positive or negative) and size of the loadings.

**Table 4:** Loadings with bootstrapped 95% confidence intervals for the first two principal components (PC).

	First PC (42%)			Second PC (21%)		
		
CAM categories	lci*	Loading	uci**	lci	Loading	uci
Alternative medical systems	-0.09	0.23	0.41	-0.71	-0.64	-0.26
Biologically-based therapies	0.35	0.44	0.52	-0.25	0.27	0.41
Energy therapies	0.15	0.38	0.52	0.05	0.49	0.66
Manipulative & body-based therapies	0.42	0.49	0.53	-0.24	-0.01	0.30
Mind-body interventions	0.25	0.42	0.53	-0.47	-0.08	0.37
Spiritual/Health literature	-0.14	0.20	0.39	-0.46	0.29	0.67
Treatment centers	0.06	0.39	0.49	-0.67	-0.44	-0.09

* lci = lower confidence interval **uci = upper confidence interval

### Relationship between CAM categories

**Figure 2:**
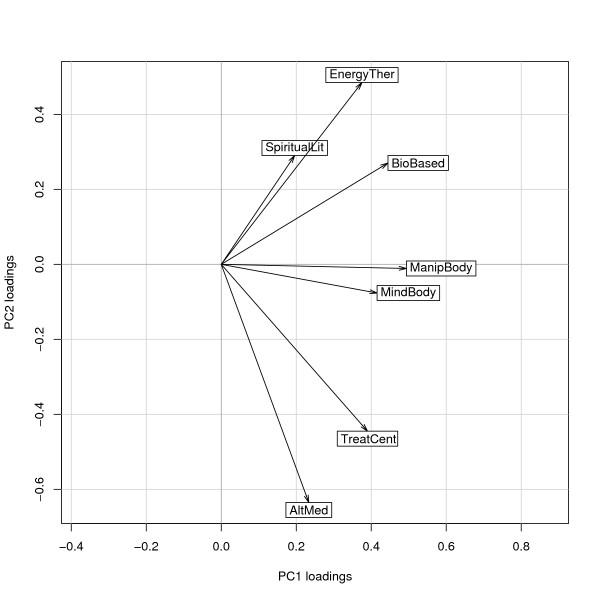
**The loadings of the original variables (i.e. number of treatments in each category) for the first two principal components**. The weights of the categories are shown in a scatter plot. See also Table 4.

Based on the loadings in Table 4, a graphic approximation of correlations between CAM categories is presented in Figure 2. CAM categories are shown as vectors where a small angle between vectors represents a strong correlation between categories and an orthogonal angle represents independence between categories. Consequently, we find that the seven categories can be grouped into three pairs and one singleton: a) *Energy Therapies *is paired with *Spiritual/Health Literature*, b) *Manipulative and body-based therapies *with *Mind-body interventions*, and c) *Alternative medical systems *with *Treatment centres*. The category *Biologically-based therapies *stands alone, located between pairs a) and b), with approximately equal positive correlations with each. We also find that the category pairs a) and c) are almost orthogonal, suggesting that the use of therapies from these categories is almost uncorrelated.

### Individual user profiles

**Figure 3:**
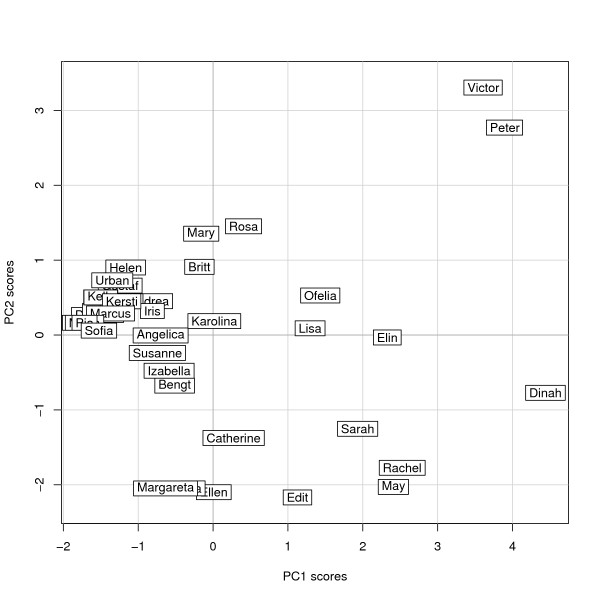
**User profiles of the study participants**. These are represented as their scores along the first two principal components, labelled by their fictitious name.

The scores of the first two PCs calculated for each user are shown in Figure 3, with the x-axis representing PC1 (number of CAM categories used) and y-axis PC2 (CAM category preference). The origin corresponds to the average user profile, i.e. a hypothetical user with average number of therapies per category and average category preference (Table 5, column 1). In Figure 3, Karolina is the study participant whose usage profile is closest to the hypothetical average user in terms of number of therapies reported, while Sofia and Dinah exemplify extremes in how few therapies and how many therapies are used, respectively. In the same manner, a large positive coordinate for PC2 indicates a stronger preference for treatments at the *Energy therapies *and *Spiritual/Health literature *end of the spectrum, whereas a high negative coordinate indicates a stronger than average preference for treatments from the categories *Alternative medical systems *and *Treatment centers*. We find e.g. that Mary, Ellen, and Karolina are similar in that they use close to the average number of therapies (close to zero on the x-axis) but have different preferences for category type. Karolina shows no particular preference for CAM category (close to zero on the y-axis), while Mary and Ellen fall at the opposite ends of the category preference axis.

**Table 5:** Four clusters of rough user patterns.

	Whole set	Cluster A	Cluster C	Cluster B	Cluster D
	Number of participants				

	n = 38	n = 24	n = 7	n = 5	n = 2

CAM categories	Average number of treatments per category				

Alternative medical systems	0.3	0.0	0.6	1.0	0.0
Biologically-based therapies	3.0	1.1	6.1	1.0	20.0
Energy therapies	0.9	0.6	1.4	0.2	5.0
Manipulative &body-based therapies	0.4	0.1	1.3	0.2	1.0
Mind-body interventions	1.3	0.7	3.0	1.4	3.0
Spiritual/Health literature	0.8	0.8	0.9	0.2	2.0
Treatment centers	0.5	0.2	1.3	1.0	0.5
PC1	0.0	-1.0	2.3	0.1	3.7
PC2	0.0	0.4	-0.7	-1.9	3.0
Representative participant	Karolina	Andrea	Sarah	Ellen	Peter, Victor

The table shows number of participants, number of average treatments per category, average value for PCs, and participants with close to average user profile for the whole data set and for the four clusters A-D.

### Groups of user profiles

**Figure 4:**
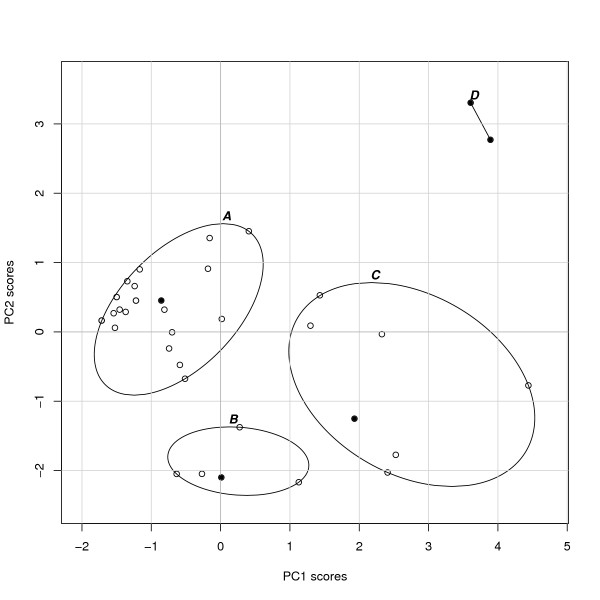
**A four cluster grouping of the user profiles**. Circles indicate individual user profiles as in Figure 3, ellipses the clusters. Full circles indicate for each cluster the subject closest to the average user profile within the group. See also Table 5.

The user profiles displayed in Figure 3 are not equally distributed, but fall into several groups. After systematically considering different cluster analysis alternatives, we suggest an interpretation based on four clusters of user patterns, displayed in Figure 4. Among these clusters, Cluster A is the largest, containing 63% of all reports, with 24 of the 38 cases (Table 5, which also shows the average number of therapies in each category). Cluster A is characterized by a preference towards the *Energy therapies *end of the spectrum, coupled with less than average use of therapies. Cluster B, with 13% of the participants, is characterized by an average number of therapies used with preference towards the categories *Alternative medical systems *and *Treatment centers*. Cluster C, with 18% of participants, is characterized by use of more than the average number of therapies with a preference towards therapies included in the categories *Alternative medical systems *and *Treatment centers*. Cluster C is the most heterogeneous of the four clusters in Figure 4, with considerable variation along both axes. Cluster D finally includes only two individuals and is characterized by the use of therapies across all CAM categories, although with a distinct preference for the categories at one end of the spectrum. This preference seems to be driven by the high number of *Biologically-based therapies *used by both individuals.

## Discussion and Conclusion

This study is an attempt to disentangle and further understand some of the variability involved in descriptions of CAM use. Based on users' own accounts, we have categorized CAM use and identified patterns in this use among a group of individuals with reported exceptional experiences in relation to cancer and CAM use. By analyzing users' own descriptions of CAM in relation to the most commonly used predefined taxonomy (i.e. NCCAM categories), this study highlights discrepancies between user and professional conceptualizations of CAM not previously addressed in other utilization studies. Beyond variations in users' reports of type and number of CAM, the explorative statistical analysis indicates some patterns in CAM usage. We suggest that preference for different CAM categories may exist along a spectrum. In this data set, reported use of therapies within the categories *Alternative Medical Systems *and *Treatment Centers *are at one end of this spectrum, while use of therapies within the category *Energy therapies *is at the opposite end of the spectrum. Although these results can not be extrapolated to CAM users in general, the patterns found in this study generate hypotheses worthy of further exploration.

### Use of a large number of CAM

The descriptive part of this analysis raises the question if use of large numbers of CAM therapies may be a feature of situations in which BHC treatments are no longer a curative option. While it is interesting to note that in contrast to many CAM utilization surveys [1], a large portion of participants in this study reported not completing BHC treatments. While we cannot draw clear conclusions from this exploratory material, this finding calls for further investigation of the relationship between adherence to and/or completion of BHC treatment and CAM use. Moreover, in line with earlier research [8,22], these results suggest a need for further exploration of the relationship between disease stage and CAM use beyond that possible with this data set.

### Discrepancy between users' descriptions of CAM and current classification systems

As noted above, these findings point to a discrepancy between NCCAM's professionally-derived taxonomy and these users' descriptions of what constitutes CAM, as the five original NCCAM categories did not satisfactorily represent participants' descriptions of the entire CAM spectrum. The categories empirically-derived from participant descriptions, *Spiritual/health literature *and *Treatment centers*, suggest a broader and less technical view of the CAM field. This expanded view is in line with the Cochrane Collaboration's definition [2] that encompasses not only practices, but also their underlying theories and belief systems.

The data in this study is limited to a brief description of these two additional categories, and further exploration of CAM users' views on therapies within these categories is warranted. Conceptualizing self-help literature as a form of CAM has previously been suggested by Achilles [23]. With the increasing information flow in society, it is important to further explore these and other mass-medial influences on patients' treatment choices.

Since it is necessary to be aware of contextual differences in what is considered CAM by different stakeholders in different societies, we suggest that these results may best serve as a basis for further discussion on appropriate development of CAM categorization to better accommodate users' descriptions of the field rather than as a suggestion for revision of NCCAM's taxomy for global use. Increased understanding of discrepancies between current professional CAM categorization and user descriptions may be crucial to improve communication and collaboration between CAM users and their providers. Moreover, knowledge about such discrepancies may help caregivers and health care organizations to acknowledge patients' views on CAM when, for example, designing integrative cancer care, since these data suggest that the environment and not only the modalities used may have therapeutic relevance.

### Challenges with heterogeneity in CAM use

While many CAM researchers argue for the importance of BHC professionals being knowledgeable about CAM [10], these results make evident some of the challenges involved. Given the large number and the wide variety of therapies used by even this small number of individuals, it is unreasonable to expect practitioners to be familiar with all possible CAM options. A major challenge lies in how to distill the most essential information about CAM therapies in general, and to find ways for both practitioners and the public to obtain more specific and trustworthy information about specific CAM modalities. Distinction by CAM category may be of value in gaining more specificity about CAM use without excess detail.

### Committed CAM use

Based on the recruitment strategy employed, the level of initiative demanded for participants to actively contact the researchers, the initial analysis of the qualitative and quantitative data generated, as well as Balneaves et al's discussion of committed CAM use [9], the participants in this study are viewed as representing a degree of commitment beyond that of the average CAM user. The relatively high median number of CAM therapies reported, supports similarities to the study sample in Balneaves et al [9] who were described as committed CAM users. However, our analysis indicates that this form of committed CAM use may still vary greatly both with regard to number and type of therapies used; consequently neither number nor type of CAM therapy is a suitable single measure of commitment. Our data suggest that commitment might be characterized either by the use of a limited number of therapeutic modalities on one hand [12], or by the use of a large number of different therapies [9]. In the latter sense, commitment may refer to a stance in regard to CAM in a broad sense, rather than as a commitment to one or more specific therapeutic modalities. Little is known about the reasons behind the use of a large number of CAM therapies. Such use might be part of an established life style or may perhaps indicate that patients are seeking something not readily found.

### Patterns of CAM use

While the heterogeneity found in this study is not unique [8,9], it supports a more nuanced view of CAM use. The explorative statistical analysis points to some general trends and patterns in these participants' reported CAM use.

The indicated relationships between the different categories shown in Figure 2, can serve as guidance for further study. The contrasting relationship between the categories *Energy therapies *on one hand and *Alternative Medical Systems *and *Treatment centers *on the other, can be interpreted as either indicating a competitive relationship between categories, or as illustrating different poles of a continuum of what constitutes acceptable and available CAM in the Swedish context. Such a continuum has previously been discussed as indicative of the level of "alternativeness" [24], reflecting the diversity of CAM therapies and their differing relationship to BHC. For example, stays at an antroposophic treatment center (category *Treatment centers*) are a reimbursable complement to BHC cancer care in many regions of Sweden, whereas most therapies reported within the category *Energy therapies *are not reimbursable. This suggests that the contrasting relationship between these categories may be a reflection of their accessibility and degree of regulation within government-regulated health care plans. It is also interesting to note that specific anthroposophic therapies in isolation (e.g. category *Biologically-based therapie*s) are not subject to reimbursement, whereas the same therapies are reimburseable when provided within the treatment center. The incorporation of certain CAM therapies within the BHC system, often described as integrative medicine [25], may be one way of assuring patient safety while maintaining patient choice.

Another possible interpretation of the contrasting relationship among CAM categories is that they may represent a competitive relationship, appealing to similar needs among participants. Since previous studies have found differences in user characteristics depending on type of CAM used, the pattern found may reflect user characteristics beyond those documented in this study. Kelner and Wellman [26] for instance, found that among a sample of 300 people using four types of CAM, users of the least institutionalized therapy had high educational levels and managerial positions to a greater extent than users of more institutionalized therapies. This raises the question if the use of less institutionalized CAM therapies is related to socio-economic factors and/or particular beliefs and attitudes.

Finally, based on the results from this study showing that individuals use a large number of CAM therapies simultaneously, important questions can be raised about the external validity of studies evaluating the efficacy of single CAM modalities. Results such as these, indicating a discrepancy between user and professional classification of CAM and patterns of CAM use, may be of value in designing intervention studies that better reflect the ways CAM are actually used.

## Abbreviations

CAM: Complementary and Alternative Medicine; BHC: Biomedically-oriented health care; NCCAM: National Center for Complementary and Alternative Medicine; IQR: Inter-quartile Ranges; PC: Principal Component

## Competing interests

The authors declare that they have no competing interests.

## Authors' contributions

The overall study design was conceived by TF, JH and CT. JH and AF conducted the interviews. JH carried out the content analysis with analytic input from AF, TF and CT. AP conducted the statistical analysis, with input from with JH and CT. JH drafted the original manuscript with input from all authors. All authors have participated in revising the manuscript, and have approved the final version.

## Pre-publication history

The pre-publication history for this paper can be accessed here:


